# Acetate-encapsulated Linolenic Acid Liposomes Reduce SARS-CoV-2 and RSV Infection

**DOI:** 10.3390/v15071429

**Published:** 2023-06-24

**Authors:** Andrew R. McGill, Eleni Markoutsa, Karthick Mayilsamy, Ryan Green, Kavya Sivakumar, Subhra Mohapatra, Shyam S. Mohapatra

**Affiliations:** 1 James A. Haley Veterans Hospital, Tampa, FL 33612, USA; 2 Center for Research and Education in Nanobioengineering, Department of Internal Medicine, Morsani College of Medicine, University of South Florida, Tampa, FL 33612, USA; 3 Department of Molecular Medicine, Morsani College of Medicine, University of South Florida, Tampa, FL 33612, USA; 4 Taneja College of Pharmacy Graduate Programs, MDC30, 12908 USF Health Drive, Tampa, FL 33612, USA; kavyasivakumar@usf.edu

**Keywords:** SARS-CoV-2, RSV, COVID-19, metabolites, fatty acids

## Abstract

Emergent Coronaviridae viruses, such as SARS-CoV-1 in 2003, MERS-CoV in 2012, and SARS-CoV-2 (CoV-2) in 2019, have caused millions of deaths. These viruses have added to the existing respiratory infection burden along with respiratory syncytial virus (RSV) and influenza. There are limited therapies for respiratory viruses, with broad-spectrum treatment remaining an unmet need. Since gut fermentation of fiber produces short-chain fatty acids (SCFA) with antiviral potential, developing a fatty acid-based broad-spectrum antiviral was investigated. Molecular docking of fatty acids showed α-linolenic acid (ALA) is likely to interact with CoV-2-S, NL63-CoV-S, and RSV-F, and an ALA-containing liposome interacted with CoV-2 directly, degrading the particle. Furthermore, a combination of ALA and a SCFA-acetate synergistically inhibited CoV2-N expression and significantly reduced viral plaque formation and IL-6 and IL-1β transcript expression in Calu-3 cells, while increasing the expression of IFN-β. A similar effect was also observed in RSV-infected A549 cells. Moreover, mice infected with a murine-adapted SARS-CoV-2 (MA10) and treated with an ALA–liposome encapsulating acetate showed significant reductions in plaque-forming units present in lung tissue and in infection-associated lung inflammation and cytokines. Taken together, these results demonstrate that the ALA liposome-encapsulating acetate can be a promising broad antiviral therapy against respiratory infections.

## 1. Introduction

The severe acute respiratory syndrome coronavirus 2 (SARS-CoV-2, synonym CoV-2), which emerged globally as the coronavirus disease (COVID-19) pandemic in 2020, has now become an endemic virus, adding to the number of other similar seasonal respiratory viral infections, such as respiratory syncytial virus (RSV) and influenza. These viral infections may cause pneumonia and acute respiratory distress syndrome, paired with the immune activation of cytokine storm syndrome, often requiring mechanical ventilation and occasionally resulting in death in severe cases [1]. Patients recovering from moderate to severe respiratory infections may suffer other neurologic or vascular diseases [2,3]. Despite the availability of vaccines, to date about one-third of the population remain opposed to vaccinations. In addition, RNA viruses are prone to mutation and the new variants often change the kinetics of infection and pathology, resulting in uncertain vaccine efficacy [4,5]. A limited number of therapies that are currently approved are either moderately effective or ineffective in moderately to severely ill patients [6,7]. Hence, there is a need to develop further broad-spectrum antiviral therapies against respiratory infections.

Broad-spectrum antivirals require an understanding of the host factors that play important roles in combating the infection pathology. Interestingly, it has been estimated that 40–45% of CoV-2 cases are asymptomatic, which raises the question of which biological factors are present in the asymptomatic group that contribute to this limited symptomology and prevention of disease [8]. One of the host factors that is key to pathology from a viral infection is the correlation observed between gut dysbiosis and disease, which established a protective role of metabolic byproducts including the short-chain fatty acid (SCFA) acetate [9,10]. Recent work has shown that SCFAs produced from the gut microbiota significantly reduced the expression of ACE2 in airway epithelial cells in culture, which could also help, in part, explain the discrepancy in symptoms [11]. Additionally, acetate has been shown to ablate infection with rhinovirus (RV) and respiratory syncytial virus (RSV) in mouse models by upregulating a free fatty acid receptor 2 (FFAR2), which induced a type 1 interferon (IFN) response by RIG-I [12,13,14]. FFAR2 activation by acetate can trigger a myriad of antiviral and antimicrobial effects through stimulating pathways that upregulate type-1 IFN [12,15]. The other SCFAs can also interact with FFAR2; however, acetate has shown the selectivity for this receptor, as well as being the predominant SCFA found in peripheral blood [16,17]. Previously, supplementation of acetate in mouse drinking water was shown to reduce the effect of CoV-2 and increase antiviral responsiveness [18].

In contrast to SCFAs, the unsaturated long-chain fatty acids (LCFAs) (including omega-3 fatty acids, such as α-linolenic acid (ALA) are solely contributed by dietary sources and serve as the second major host factor with pleiotropic physiological roles including governing lipid metabolism and the regulation of inflammation. Furthermore, LCFAs have been shown to inactivate enveloped viruses [19,20]. Patients with severe cases of COVID-19 have been shown to possess lower levels of omega-3 in their blood compared to patients with less severe disease [21]. Furthermore, supplementation with omega-3 fatty acids such as ALA was effective in preventing and treating COVID-19 by reducing the risk of being positive for CoV-2 infection; it limited the duration of symptoms, had a positive effect on respiratory and renal dysfunction, and increased the overall survival rate of COVID-19 patients [22,23]. Mechanistically, omega-3 LCFAs activate FFAR4, resulting in an anti-inflammatory phenotype, as well as promoting tissue repair [24]. The consumption of omega-3 fatty acids has been shown to lower markers of inflammation in serum, such as C-reactive protein, interleukin-6 (IL-6), interleukin-1β (IL-1β), and tumor necrosis factor-α (TNF-α), and other inflammatory factors that have been implicated in the cytokine storm of COVID-19 [25,26,27]. Structural analyses revealed that ALA binds to the CoV-2 spike protein RBD pocket, antagonizing its ability to access and bind to host ACE2 [28]. In addition, ALA was shown to inhibit TMPRSS2 protease and cathepsin L activity without altering the concentration of the other proteases and ACE2 at the protein level or ACE2 activity [29,30].

RSV is another respiratory virus of significant consequence, which had a record surge, driven by multiple lineages, in the United States during the 2022 season [31]. RSV is a major respiratory pathogen and a significant pediatric developmental burden and is the main causative agent of pediatric bronchiolitis worldwide [32,33,34,35]. Improved epidemiologic data have shown RSV to be an increasingly important pathogen in the elderly and immunocompromised [36]. The incidence of disease at the extremes of age can be attributed in part to the under-developed immune systems of infants and the senescing immune systems of the elderly [36,37]. An important feature of this virus is that no long-term immunity is garnered post-infection, and there are multiple infections throughout life. Annually, in the United States, RSV is the leading cause of pediatric hospitalization, with an average of 1.6 million outpatient visits among children younger than 24 months old with 58,000–80,000 hospitalizations for severe complications for those <5 years of age, and 60,000–160,000 hospitalizations among adults ≥ 65 years [38,39]. During the acute phase, RSV presents itself like a common cold, infecting the upper and lower respiratory tract; however, there are serious complications that can arise. These complications include bronchiolitis in young children, pneumonia, apnea, respiratory failure, and the exacerbation of asthma and can be life-threatening [35]. Further complicating matters is the fact that RSV is a negative sense ssRNA virus, making it prone to mutation [40]. Additionally, there are two dominant strains of RSV that circulate globally, making a broadly applicable therapy or vaccine difficult to create, further complicated by variant generation [41]. The only approved therapy for RSV disease in pediatric cases is aerosolized Ribavirin. Also, anti-F monoclonal antibody therapies such as nirsevimab, and palivizumab are used as prophylactics for high-risk groups, such as premature infants [42,43]. However, these treatments have moderate efficacy and are expensive [42]. The standard treatment of RSV disease is supportive care until symptoms subside; however, severe RSV infections, particularly in pediatric cases, can result in the development of asthma or cognition issues later in life [42,44]. This illustrates the need for the development of a prophylactic therapy against the progression of severe disease incidence, which may comprise methods that target both viral and host factors, as is also needed for COVID-19 prevention.

The major premise of this investigation has been to develop broad-spectrum antiviral medications against common respiratory infections causing pneumonia. Previously, we reported a nanoformulation to enhance antiviral therapy against RSV by inhibiting both virus replication and virus fusion [45]. Since increased levels of gut microbiota-induced acetate in the lung and serum levels and diet-induced omega-3 fatty acid are known to be protective against both SARS-CoV-2 and RSV infections, we examined the interaction of the viral surface glycoprotein of SARS-CoV-2, Spike S1 (CoV-2 S) with different omega-class fatty acids [12,18,21,22,30,46]. This was performed by molecular docking, which led us to identify ALA as the top candidate in binding to CoV-2 S by ligand affinity score (Supplementary Figure S1A,B). We also found that the RSV fusion protein (RSV-F) and the spike protein of NL63-CoV also interacted with ALA in a similar fashion (Supplementary Figure S1A,B). The results led us to hypothesize that nanoformulating a synergistic combination of acetate as a replication inhibitor, and ALA as a fusion inhibitor may provide an effective broad antiviral treatment for respiratory infections. In this report, we delineate the results of our studies, which shows that combinations of acetate and ALA synergistically reduce respiratory viral infections caused by CoV-2 or RSV. Further, our results show that an ALA liposomal nanoformulation carrying acetate as an antiviral payload is released upon interacting with CoV-2. Additionally, we performed in vivo studies in murine-adapted SARS-CoV-2 (MA10) infected mice by treating them with ALA–acetate liposome. Furthermore, results of studies on the antiviral mechanism suggest synergy, which involves crosstalk between the receptors, i.e., acetate receptor FFAR2 and ALA receptor FFAR4, affecting both their expression and signaling functions. Taken together, our results demonstrate for the first time that a novel ALA liposomal nanoparticle (NP) with an acetate payload can act as a broad antiviral with intranasal delivery.

## 2. Materials and Methods

### 2.1. Cell Culture

Calu-3 (Clone 2B4)-ACE2 (BEI Resources, Manassas, VA, USA: NR-55340) samples were maintained at 37 °C with 5% CO_2_ atmosphere and grown in Dulbecco’s Modified Eagle’s Medium supplemented with 20% fetal bovine serum, 1% penicillin–streptomycin antibiotic, 4 mM L-glutamine, 4500 mg/L glucose, 1 mM sodium pyruvate, and 1500 mg/L sodium bicarbonate. VeroE6-ACE2-TMPRSS2 (BEI Resources, Manassas, VA, USA: NR-54970) samples were maintained at 37 °C with 5% CO_2_ atmosphere and grown in Dulbecco’s Modified Eagle’s Medium supplemented with 10% fetal bovine serum, 1% penicillin–streptomycin antibiotic, 4 mM L-glutamine, 4500 mg/L glucose, 1 mM sodium pyruvate, 1500 mg/L sodium bicarbonate, and 10 μg/mL puromycin. A549 (ATCC, Manassas, VA, USA) samples were maintained at 37 °C with 5% CO_2_ atmosphere and grown in Dulbecco’s Modified Eagle’s Medium supplemented with 10% fetal bovine serum, 1% penicillin–streptomycin antibiotic, 4 mM L-glutamine, 4500 mg/L glucose, 1 mM sodium pyruvate, and 1500 mg/L sodium bicarbonate. 3T3 cells (ATCC, Manassas, VA, USA) were maintained at 37 °C with 5% CO_2_ atmosphere grown in Dulbecco’s Modified Eagle’s Medium supplemented with 10% fetal bovine serum, 1% penicillin–streptomycin antibiotic, 4 mM L-glutamine, 4500 mg/L glucose, and 1500 mg/L sodium bicarbonate. For inhibition studies, GLPG0974 was used at 30 μM to inhibit FFAR2, and FFAR4 was inhibited using AH 7614 at 25 µM per the manufacturer’s instructions (Cayman, Ann Arbor, MI, USA). All cells were negative for mycoplasma.

### 2.2. Virus Propagation and Plaque Assays

All experiments using replication-competent SARS-CoV-2 were performed in a biosafety level 3 laboratory (BSL-3) at the University of South Florida. Viral stocks of CoV-2 were produced from infection of Vero-E6 cells expressing ACE2 and TMPRSS2 (BEI Resources, Manassas, VA, USA NR-54970), cultured in T75 flasks to a confluency of 80–90%, and infected with either MA10-SARS-CoV-2 or icSARS-CoV-2-nMG and titrated as previously described [3,47]. Viruses obtained from lung homogenates were also titrated using this method. SARS-CoV-2 nMG was a kind gift from Dr. Pei-Yong Shi from the University of Texas Medical Branch, Galveston, TX, USA [48]. MA10-SARS-CoV-2 was obtained from BEI Resources (BEI Resources, Manassas, VA, USA: NR-55329). RSV rA2-KL19F was prepared as previously described [45]. Importantly, this strain of RSV expresses RFP-mKate2, and was used to estimate the percent infectivity of cells over cells stained with NucBlue-Hoechst 33342 Reagent (Thermo, Waltham, MA, USA). Quantification was performed using ImageJ 1.x software (NIH).

### 2.3. RNA Isolation and Quantitative Polymerase Chain Reaction (qPCR)

Total RNA was isolated using Trizol (Life Technologies, Carlsbad, CA, USA) and cDNA was generated as previously described [3,47]. Quantitative real-time PCR reaction (qPCR) was performed using the cDNA generated from the extracted RNA using a CFX384 TouchTM Real-Time PCR detection system (Bio-Rad, Hercules, CA, USA). The reaction mixture was set up to a total of 5 µL containing 1 µL of 5× qPCR master mix, 0.5 µL of forward primer and reverse primer,1 µL of water and 1 µL of cDNA. The reaction was performed using the following program: 95 °C or 3 min, followed by 45 cycles of 95 °C for 10 s, 60 °C for 1 min, and 72 °C for 15 s. Samples were ran in quadruplicate and calculated. A table lists qPCR primers below; mm = mus musculus, hu = human. (See Supplementary Table S1).

### 2.4. Liposome Formulation

Liposomes were generated using the thin-film hydration method [49]. A ratio 6:3:1 of Egg-PC (Avanti, Alabaster, Al, USA): α-linolenic acid (Sigma, St. Louis, MO, USA): cholesterol (Sigma, St. Louis, MO, USA) was used. Liposomes were hydrated with a 150 mM acetate solution in water and then sonicated. Particles were dialyzed overnight and analyzed by dynamic light scattering (Malvern, Malvern, UK) the next day. Acetate encapsulation efficiency was determined by quantitative acetate assay (Sigma, St. Louis, MO, USA). Phosphate-buffered saline was used in place of acetate for control liposome generation. Further, vehicle control liposomes contained only Egg-PC and cholesterol and encapsulated 1× PBS or 150 mM acetate. To hydrate the lipid layer, 100 μM calcein was used to form calcein-encapsulated liposomes for release studies [50,51]. ALA-calcein particles were ran through a Sephadex column to remove unencapsulated calcein. DIR (1,1′-Dioctadecyl-3,3,3′,3′-Tetramethylindotricarbocyanine Iodide) (Thermo, Waltham, MA, USA) was incorporated into the ALA-Acetate liposomal nanoformulation for biodistribution studies at the lipid film generation step at a ratio of 0.2, all other components were added at their previously mentioned ratios and concentrations, and hydrated with 150mM acetate solution [52]. Lipids were quantified using the Stewart assay as previously described [53].

### 2.5. Calcein Release

For measuring calcein release, 300 µL of 400 mg/mL liposome, with or without ALA, encapsulating calcein, was incubated at room temperature with 3.0 × 10^6^ pfu of UV inactivated CoV-2-WT or MA10. The incubated mixture was time-interval sampled and analyzed by Synergy H4 Hybrid Reader (BioTek, Winooski, VT, USA) at 495 nm excitation and 515 nm emission. To rupture all liposomes after the plate was read, 2% triton (Sigma, St. Louis, MO, USA) was added to the reaction, the sample was mixed, and the plate was read again to show the maximum calcein present in the sample, which was used for normalization.

### 2.6. Animal Experiments

All animal procedures were conducted in accordance with the NIH guidelines for the Care and Use of Laboratory Animals and approved by the Institutional Animal Care and Use Committee of the University of South Florida (Protocol IS00008091 “Screening Antivirals for COVID-19”, approved 20 January 2021). Ten-week-old male Balb/c mice were housed in a BSL-3 animal facility and were infected with 1.25 × 10^5^ pfu of CoV-2-MA10 as previously described [3,47]; organs were collected and processed as described previously. For biodistribution of DIR-ALA liposomes, ten-week-old male Balb/c mice were deeply sedated under isoflurane anesthesia and inoculated with 5 × 10^5^ pfu UV inactivated CoV-2 1 h before 50 µL intranasal administration of liposomes under anesthesia. Mice were observed to fully recover from the effects of anesthesia. Ex vivo imaging was conducted using the IVIS^®^ Spectrum in vivo imaging system (PerkinElmer, Waltham, MA, USA). Acetate concentrations in the blood and lungs of liposome-inoculated mice were determined by Acetate Colorimetric Assay Kit (Sigma, St. Louis, MO, USA).

### 2.7. Histology

Lungs were fixed in formalin, then dehydrated and embedded in paraffin as previously described [54]. Lungs were sectioned at 10 μM and stained using H&E. IHC was performed according to standard protocols, and lung tissues were stained with FITC-conjugated FFAR2, according to the manufacturer’s instruction (Fisher, Hampton, NH, USA). After staining, FFAR2–FITC-stained slides were fixed with a permeant containing DAPI (Fisher, Hampton, NH, USA).

### 2.8. Statistical Analysis

All data are presented as mean ± standard deviation (S.D.). Experiments were conducted with a minimum of three biological replicates. Data from representative experiments are shown. Statistical significance was evaluated by ANOVA and Students *t*-test. A *p*-value of less than 0.05 was considered statistically significant for all comparisons.

## 3. Results

### 3.1. Acetate and ALA Act Synergically In Vitro against CoV-2 and Reduce COVID-19-Associated Cytokines

**Figure 1:**
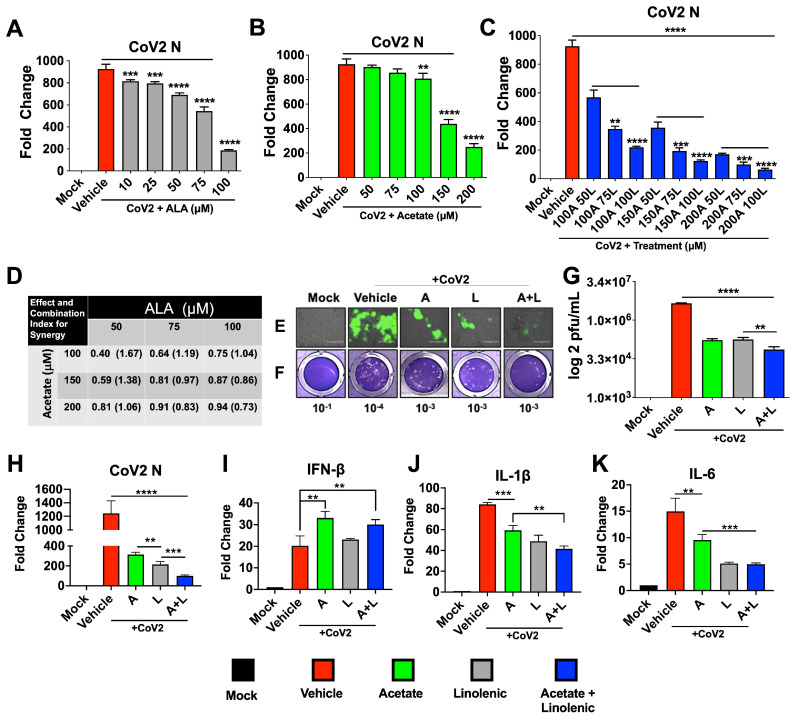
Treatments with a combination of acetate and ALA increase the reduction of viral infection. Calu3 cells were infected with SARS-CoV-2 at 0.1 MOI for 24 h prior to treatment with varying concentrations of acetate (A) and/or linolenic acid (L). (**A**–**C**) qPCR analysis of N-gene expression following treatment with increasing concentration of L or A, or in combination, normalized to β-actin. (**D**) Combination index (CI) and synergy were determined by the generation of a combination index plot using CompuSyn software. The CI values are based on the equation for multiple drug effect interactions and are used for quantitative determinations of synergism (CI < 1), additive effect (CI = 1), and antagonism (CI > 1). (**E**–**K**) Treatment with 200 μM A and/or 100 μM L decreases infection, as assessed by immunofluorescent imaging (20× magnification) at 72 hpi (**E**). Plaque assay from supernatants of treated cells overlaid on VeroE6-ACE2-TMPRSS2 cells, showing representative plaque images (**F**). Mean plaque assays conducted with 2 replicates from supernatants of infected samples, and significance was determined using a *t*-test, ±SD (**G**). Gene expression of SARS-CoV-2 N and human cytokines IFN-β, IL-1β, and IL-6 analyzed by qRT-PCR, normalized to β-actin (**H**–**K**). All experiments were conducted in 3 biological replicates. Data from representative experiments are shown. For qPCR, significance was determined using a one-way ANOVA with multiple comparisons, ±SD, ** *p* < 0.01, *** *p* < 0.001, **** *p* < 0.0001.

Toward our search for a broad antiviral targeting respiratory viruses, we undertook molecular docking analysis of viral surface proteins such as the CoV-2 S, NL63-S, and RSV F1 fusion protein with a comprehensive panel of free fatty acids and found that the ALA had the highest docking score (Supplementary Figure S1) followed by linoleic acid (not shown). Docking studies also showed that both ALA and linolenic acid interact with CoV-2 S at the Arg 403, Gly 404, Asp 405, and Glu 406 residues, although ALA binds more strongly to Arg 403, resulting in a higher ligand affinity score and suggesting that ALA may act as a potent fusion inhibitor. To test the inhibitory role of ALA, we infected Calu-3 cells with 0.1 multiplicity of infection (MOI) CoV-2 and treated them with ALA the next day. ALA treatment showed a dose-dependent response in reducing infection, as shown by qPCR for the CoV-2 nucleoprotein (N) gene expression analysis (Figure 1A). Acetate is known to have antiviral activity, modulating type-1 IFNs and thereby decreasing viral replication [14,30]; however, pretreatment with acetate, which has a short half-life [11,14,55,56] showed a dose-dependent response in CoV2-infected Calu-3 cells at concentrations in the range of 100–200 µM. We reasoned that acetate in combination with ALA can synergize to create a more potent broad-spectrum antiviral. Thus, infected cells were treated with increasing concentrations (in the range of 100–200 µM) of acetate and ALA (in the range of 50–100 µM), and the combination index for synergy was assessed using CompuSyn, version 1.0 (Figure 1A–D) [57]. qPCR analyses of RNA at 72 h post-infection (hpi) showed synergistic reductions in CoV-2 N gene expression. The lowest (best) synergy score of 0.73 was found to be at 200 µM acetate with 100 µM ALA from the concentrations assayed (Figure 1D). This combination was used in downstream work to assess the phenotypic effect on CoV-2 infection. Previous work by our group and others demonstrated that acetate concentrations have therapeutic efficacy against respiratory viruses at 200 µM, whereas the LCFAs have efficacy at 100 µM [12,30]. This synergistic combination of acetate and ALA significantly decreased the expression of mNeonGreen (mNG) encoded within the CoV-2 nMG genome (Figure 1E), viral titers, as determined by plaque assay (Figure 1F,G), and N-gene expression (Figure 1H). Furthermore, this synergistic combination of acetate and ALA significantly increased the expression of host interferon-β (IFN-β) and decreased the expression of host interleukins IL-1β and IL-6, which are associated with severe COVID-19 (Figure 1I–K).

### 3.2. The Antiviral Roles of Acetate and ALA Involve Their Action through Their Respective Receptors

**Figure 2:**
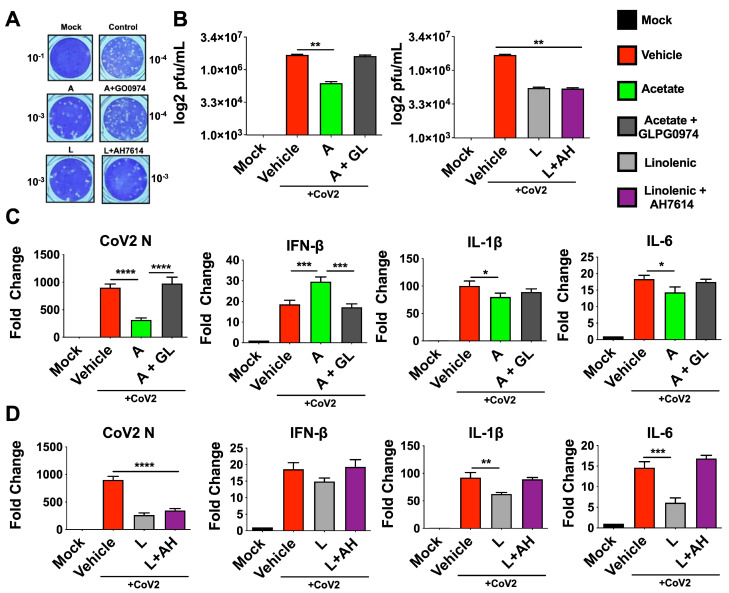
Inhibition of FFAR2 and FFAR4 partially reverses the antiviral phenotype. Calu3 cells were pre-treated with either 30 μM GLPG0974 (GL) or 25 μM AH 7614 (AH) and subsequently infected with 0.1 MOI SARS-CoV-2 nMG 24 h before treatment with 200 μM acetate (A) or 100 μM linolenic acid (L). (**A**,**B**) Plaque assay from supernatants of treated cells (72 hpi) overlaid on VeroE6-ACE2-TMPRSS2 cells, showing representative plaque images (**A**). Mean plaque assays conducted with 2 replicates from supernatants of infected samples, and significance was determined using *t*-test, ±SD (**B**). (**C**,**D**) qRT-PCR for SARS-CoV-2 N and human IFN-β, IL-1β, and IL-6 from cells treated with A or L, with results normalized to β-Actin. All experiments were conducted in 3 biological replicates and all plaque assays were conducted with 2 replicates from supernatants from infected samples. Data from representative experiments are shown. For qPCR, significance was determined using a one-way ANOVA with multiple comparisons, ±SD, * *p* < 0.05, ** *p* < 0.01, *** *p* < 0.001, **** *p* < 0.0001.

FFARs are G-protein-coupled receptors that have potent physiological effects on their hosts [58,59,60]. FFAR2 is the receptor for SCFA-acetate and is shown to upregulate antiviral factors including type-1 IFN [12,61]. On the other hand, FFAR2 and FFAR4 have been shown to downregulate Th2 inflammatory pathways implicated in cytokine storm in severe COVID-19 cases, including inflammatory cytokines IL-1β and IL-6 [12,62,63]. These two FFARs are also known to be expressed in the lung epithelial cells and other immune cells [59,60,64,65,66]. To examine the basis of their antiviral roles in lung-derived Calu-3 cells, we used inhibitors of receptors that are known to bind to these free fatty acids [67,68]. Thus, cells were pretreated with inhibitors and infected, then treated with acetate or ALA 24 hpi. Supernatants from this experiment show an increase in plaques in accordance with treatment with GLPG0974 (an inhibitor of FFAR2) in the presence of acetate compared to acetate treated cells without the inhibitor. However, treatment with AH7614 (an inhibitor of FFAR4) showed no significant change in plaque count compared to ALA treatment (Figure 2A,B). Further, results showed that treatment with GLPG0974 reversed the protective effects of acetate, as seen by increased N, IL-6, and IL-1β gene expression and decreased IFN-β mRNA expression (Figure 2C). In contrast, treatment with AH7614 suggested that the antiviral effect of ALA was independent of the FFAR4 interaction, given that there was no effect on titers or N expression; however, IL-1β and IL-6 expression were reversed when FFAR4 was inhibited (Figure 2D). The N-gene expression in inhibitor-treated cells paralleled the viral titers as determined by plaque assay.

### 3.3. Acetate and ALA Reduces RSV Encoded RFP Expression in Infected A549 Cells

**Figure 3:**
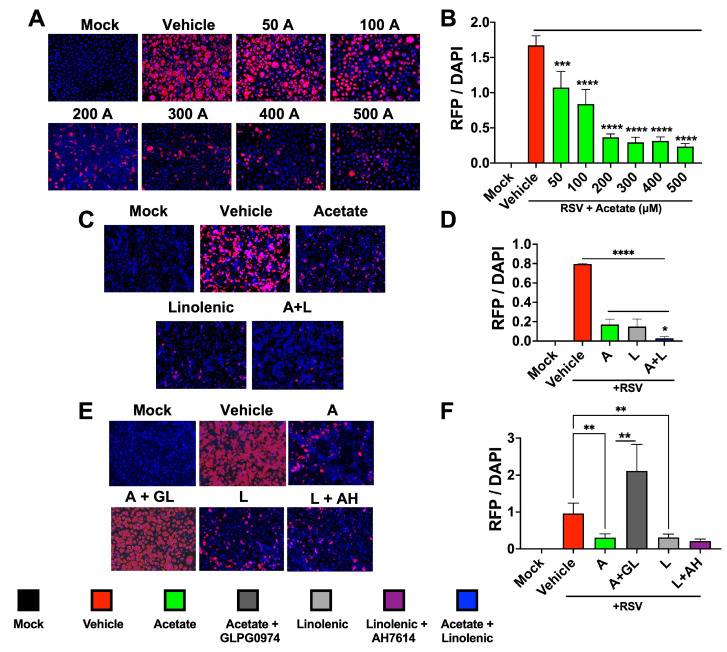
Combination of acetate and ALA reduces RSV infection. (**A**) A549 cells were infected with rA2-KL19F at 3 MOI and treated the next day with 50, 100, 200, 300, 400, or 500 μM acetate (A). (**B**) Cells were imaged 48 h post-infection at 20× and the integrated density of RFP/DAPI fluorescence from RSV infected A549 cells was quantified using ImageJ. (**C**) A549 cells were infected with rA2-KL19F at 3 MOI and treated the next day with 200 μM acetate (A), 100 μM linolenic acid (L), or a combination (A + L). (**D**) The integrated density of RFP/DAPI fluorescence from RSV infected A549 cells from C, quantified using ImageJ. (**E**) A549 Cells pre-treated with either 30 μM GLPG0974 (GL) or 25 μM AH 7614 (AH) were then infected with K19-RSV Red at 3 MOI and treated the next day with either 200 μM acetate or 100 μM linolenic acid. (**F**) The integrated density of RFP/DAPI fluorescence from RSV infected A549 cells from **E**, quantified using ImageJ. All experiments were conducted in 3 biological replicates. Data from representative experiments are shown. Significance was determined using a one-way ANOVA with multiple comparisons, ±SD, * *p* < 0.05, ** *p* < 0.01, *** *p* < 0.001, **** *p* < 0.0001.

Our molecular docking analysis of the RSV-F1 and LCFAs showed that omega-fatty acids interact with RSV-F (Supplementary Figure S1). Similarly to CoV-2, a dose response for acetate was seen in RSV-infected A549 cells using rA2-KL19F RSV, which expresses red fluorescent protein (RFP) mKate2 with significant reductions in fluorescence (Figure 3A,B). Effects of supplemented acetate in both in vitro and in vivo models [12] and our results from CoV-2 infections prompted us to investigate whether acetate could impact RSV infection in the presence of ALA (Figure 3C,D). Interestingly, the combinations that were used against CoV-2 were also effective in reducing RFP mKate2 marker expression encoded by RSV in treated A549 cells, indicating that this treatment may also show an additive effect against RSV infection and/or replication (Figure 3C,D). To test if the FFARs were also impacting this phenotype, A549 cells were infected with rA2-KL19F in the presence of GLPG0974 and AH7614 overnight and treated the next day with acetate, ALA, or a combination. Similarly to the CoV-2 results, inhibition of FFAR2 increased the expression of RFP in RSV-infected cells, reversing the protective phenotype induced by acetate (Figure 3E,F). However, the presence of the FFAR4 inhibitor had no effect on RFP expression, suggesting that inhibition of RSV infection by ALA is independent of FFAR4 binding and signaling. These results indicate that the combination of acetate and ALA can provide effective therapy for RSV infections.

### 3.4. ALA–Liposomal NPs Interact with CoV-2, and Are Retained in the Lungs of Mice

**Figure 4:**
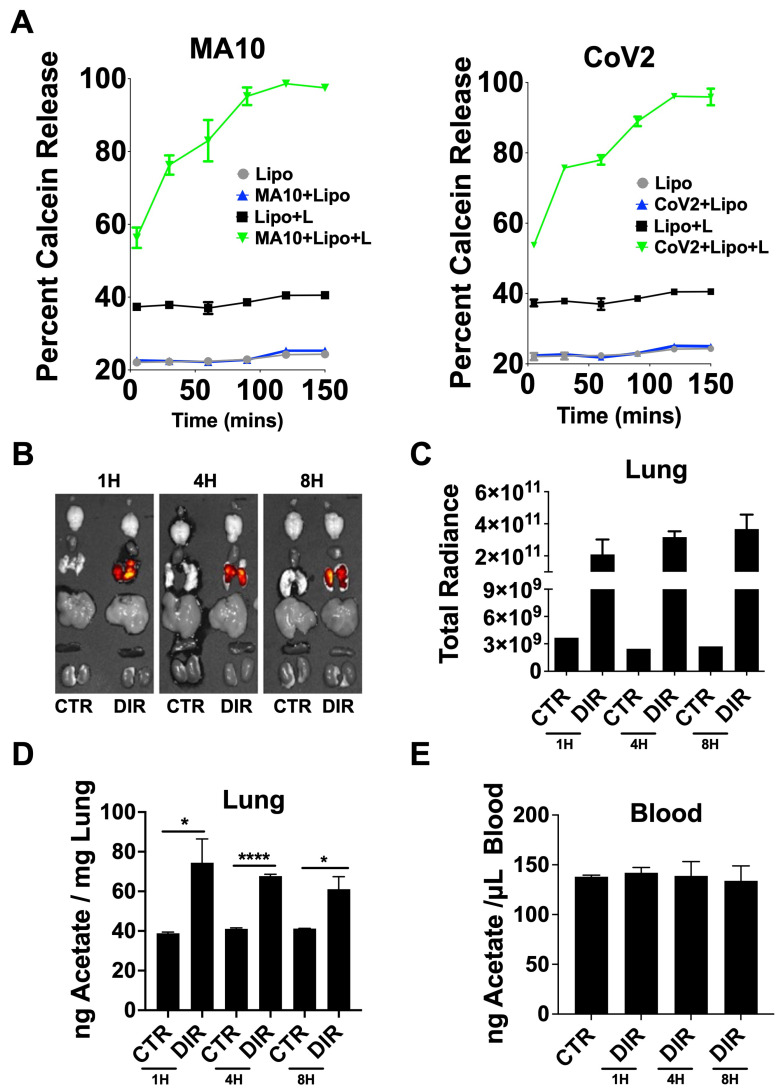
Bioavailability and biodistribution of DIR–ALA–acetate liposomes. (**A**) Calcein release from linolenic–liposomes (Lipo + L) incubated with UV inactivated MA10 or WT-CoV-2. (**B**) DIR fluorescence ex vivo representative images of mouse organs from IVIS^®^ Spectrum at 1, 4, and 8 h after particle administration, compared to control (CTR). (**C**) Total radiant efficiency of lungs from liposome-inoculated mice compared to control. Total radiant efficiency calculated as [p/s]/[µW/cm^2^]. (**D**) Nanogram of acetate per mg of lung homogenate from DIR-ALA-acetate-liposome inoculated mice quantified using the acetate colorimetric assay kit (Sigma-Aldrich, St. Louis, MO, USA). (**E**) Acetate concentrations in the blood of DIR-ALA-acetate-liposome-inoculated mice. Lung and blood acetate was quantified using the acetate colorimetric assay kit (Sigma-Aldrich). All experiments were conducted in 3 biological replicates. Data from representative experiments are shown. Significance was determined using *t*-test, ±SD, * *p* < 0.05, **** *p* < 0.0001.

To investigate whether ALA directly interacts with the CoV-2 virus, we designed and synthesized a liposomal NP using ALA as a lipid. Thus, phospholipids, ALA, and cholesterol were formed into liposomes using the thin-film hydration method. The film was hydrated with acetate in PBS and particles were characterized for their size (Supplementary Figure S2). A cell viability assay using 3T3 and A549 cells was also conducted, and no toxicity was seen, consistent with what others have reported [69,70] (Supplementary Figure S3). Calcein dye was used in place of acetate, as a surrogate, to test in vitro release in the presence of UV-inactivated- wild-type CoV-2 (WT-CoV-2) and a murine adapted strain (MA10). Inactivated virus administration triggered a release in calcein that was detected using 495 nm excitation/515 nm emission at specified time points (Figure 4A). Calcein contained in control phospholipid liposomes lacking ALA did not release; however, the ALA–liposomal NPs yielded a release of calcein upon interaction with inactivated WT-CoV2 or MA10. This interaction highlights how these novel particles formulated with ALA bind CoV-2 with particle degradation occurring upon viral interaction (Supplementary Figure S1C) releasing the payload.

To investigate the stability of these NPs in vivo, acetate-encapsulated ALA–liposomal NPs were co-formulated with DIR, a lipophilic near-infrared fluorescent dye, displaying excitation/emission maxima of 750/780 nm, respectively, for tracking of liposomes. Mice had 5 × 10^5^ pfu of UV-inactivated MA10 intranasally administered 1 h prior to liposome treatment over a time course of 8 h, with biodistribution determined using ex vivo imaging (Figure 4B,C). The DIR signal remained elevated in the lungs, indicating that the lipid formulation also likely remained in the lungs due to no signal being seen in other organs collected. Lung homogenate and serum were assayed for acetate using the colorimetric acetate assay kit and compared to mock infected mice. Similar to the DIR signal results, acetate remained elevated in the lungs 8 h post-treatment with DIR-ALA-acetate liposome, with no change detected in serum between groups (Figure 4D,E).

### 3.5. Administration of Acetate-encapsulated ALA-Liposomal NPs Reduces MA10 Infection and Associated Cytokines in a Murine Model

**Figure 5:**
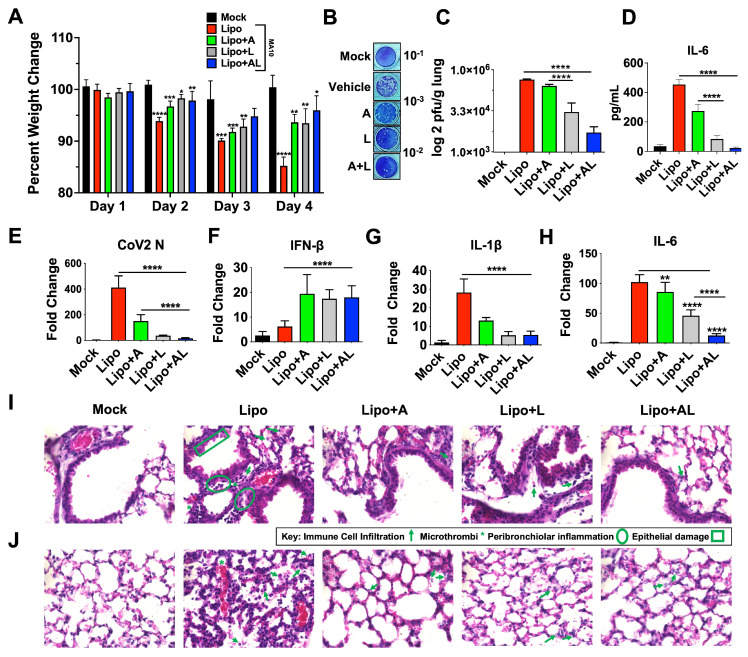
Treatment of MA10 infected with ALA liposomes ablates infection and COVID-19 associated cytokines. (**A**) Percentage weight change of mice infected with MA10 and treated with either blank liposome (Lipo), acetate–liposome (Lipo+A), ALA–liposome (Lipo+L), or acetate–ALA–liposome (Lipo+AL). (**B**,**C**) Lung homogenates were used for plaque assay on VeroE6 ACE2-TMPRSS2 cells, showing representative plaque images (**B**), mean plaque assays conducted with 2 replicates from supernatants of infected samples; significance was determined using *t*-test, ±SD (**C**). (**D**) ELISA was used to quantify IL-6 in serum. (**E**–**H**) qRT-PCR for SARS-CoV-2 N, mouse IL-1β, IL-6, and IFN-β, with results normalized to β-Actin. (**I**,**J**) Representative images of 10 um paraffin embedded H&E-stained lung sections of bronchiole (**I**) and alveoli (**J**) shown at 60×. All experiments were conducted in 4 biological replicates. Two sections per mouse were analyzed. Data from representative experiments are shown. Plaque assay samples were pooled and ran in duplicate, per biological replicate. Significance was determined using a one-way ANOVA with multiple comparisons, ±SD, * *p* < 0.05, ** *p* < 0.01, *** *p*< 0.001, **** *p* < 0.0001.

To investigate whether acetate and/or ALA liposomal NPs reduce infection in vivo, we used a murine-adapted SARS-CoV-2 (MA10) [71], which was demonstrated to induce acute lung injury and mortality similar to COVID-19 in humans. To test the efficacy of acetate or/and ALA Liposomal NPs, 10-week-old male BALB/c mice were intranasally inoculated with 1.25 × 10^5^ pfu of MA10. Mice were treated after 24 h of infection and once daily for 3 days, and then sacrificed on day 5. Body weight taken for the duration of the experiment showed that the mice that received liposomes with acetate and ALA significantly maintained a greater percentage of weight compared to either one of them alone or the control (Figure 5A). On termination, the lungs were processed for plaque assay, histological analysis, and qPCR. Mice treated with ALA–acetate liposomal NPs showed significant reductions in viral titers in their lung homogenate compared to those treated with ALA or acetate alone or liposomal control (Figure 5B,C). Additionally, serum IL-6 levels examined by ELISA showed a decrease in IL-6 for all treated groups compared to control (Figure 5D). Furthermore, qPCR of lung homogenates showed a decrease in CoV-2 N expression, increased IFN-β expression, and a decrease in host inflammatory cytokines IL-6 and IL-1β compared to control (Figure 5E–H). Lung histological analysis using hematoxylin and eosin (H&E) staining showed less bronchial, peribronchiolar, and alveolar inflammation as well as less epithelial damage, immune cell infiltration, and microthrombi in the groups treated with ALA liposomes containing acetate compared to the lipo control, (Figure 5I,J). Taken together, these data show that ALA–acetate liposomal NPs reduce viral titers in the lung while simultaneously upregulating the expression of a type-1 IFN cytokine and dampening host inflammatory cytokines and preserving the architecture of the lung.

### 3.6. The Synergy between Acetate and ALA Involves Significant Upregulation of FFAR2 and FFAR4 Expression

**Figure 6:**
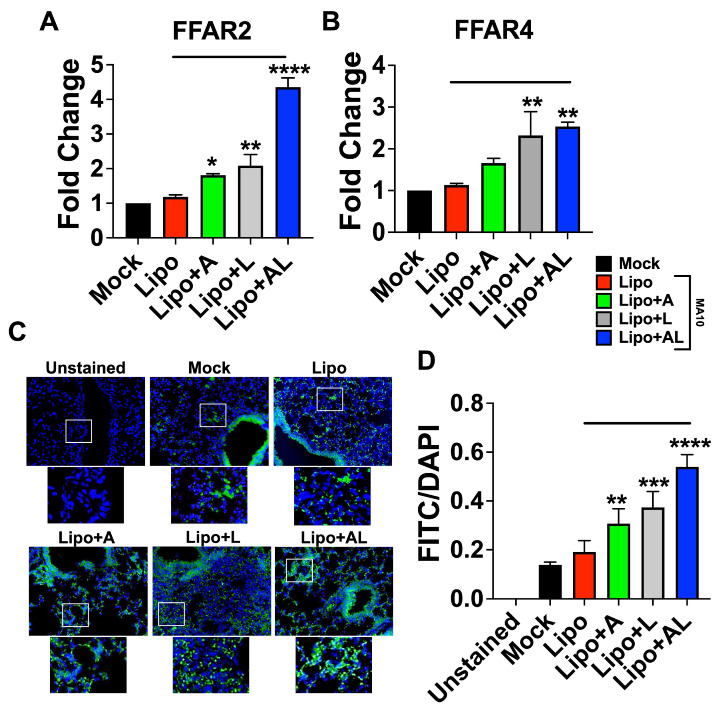
FFAR2 and FFAR4 are upregulated by ALA–acetate liposome treatment in SARS-CoV-2 MA10 infected mice. (**A**,**B**) FFAR2 and FFAR4 gene expression by qPCR from RNA isolated from lung homogenate from in vivo experiment in Figure 5; results normalized to β-actin. (**C**) Representative images of 10 μm sections from paraffin-embedded lungs processed for IHC and stained with anti-FFAR2 antibody conjugated to FITC, imaged at 40×, 3 biological replicates. (**D**) Plotted integrated density of FITC and DAPI fluorescence from anti-FFAR2 stained lung sections. All experiments were conducted in 3 biological replicates. Data from representative experiments are shown. Significance was determined using a one-way ANOVA with multiple comparisons, ±SD, * *p* < 0.05, ** *p* < 0.01, *** *p* < 0.001, **** *p* < 0.0001.

Since receptors FFAR2 and FFAR4 are expressed by epithelial cells and immune cells including macrophages, we reasoned that synergy between acetate and ALA may result from upregulation of FFAR2 and FFAR4, with downstream effector molecules having a potential molecular crosstalk (Supplementary Figure S5). To test this hypothesis, we examined the expression of FFAR2 and FFAR4, which are responsible for the dominant antiviral and anti-inflammatory effects, respectively. The results of mRNA expression of FFAR2 by qPCR showed that treatment of mock- and virus-infected mice with the combination of acetate and ALA significantly increased FFAR2 expression, compared to treatment with either of them alone (Figure 6A). The results were further verified by FFAR2 protein antigen-expression analysis in infected mice treated with liposomes containing acetate or/and ALA using immunohistochemistry on paraffin-embedded lung sections from the experiment in Figure 5. The results showed a significantly increased staining of FFAR2 in these lung sections in the acetate-encapsulated ALA liposome treated group compared to other groups (Figure 6C,D). The increased expression of FFAR2 in the lungs upon treatment with an acetate-encapsulated ALA liposome group indicates that the host response to these fatty acid combinations may directly promote greater antiviral responsiveness through the upregulation of FFAR2. Increased FFAR4 mRNA expression was seen in mice treated with ALA liposome or the combination suggesting no involvement of acetate in modulating FFAR4 expression (Figure 6B). 

## 4. Discussion

Overall, the results of these studies demonstrate for the first time that a combination of short- and long-chain fatty acids, namely acetate and ALA, synergistically reduce respiratory viral infections caused by CoV-2 or RSV both in vitro and in vivo. Further, our results show that ALA liposomal nano-formulation can carry acetate as an antiviral payload, which is released upon interaction with CoV-2. Results of studies on the antiviral mechanism of acetate-ALA NP suggest that the synergy between the SCFA and LCFA involves cross-regulation between acetate receptor FFAR2 and ALA receptor FFAR4, leading to increased expression of FFAR2 and downstream signaling functions.

One of the major findings of this report is the synergy between two host-derived factors, acetate and ALA. Firstly, host microbiome-controlled metabolic products, including acetate, are known to inhibit viral replication in several respiratory viral infections by the upregulation of type-I IFNs [12,13,14,18]. However, infection-induced gut dysbiosis can limit the level of acetate in the lungs and the short half-life of free acetate limits its therapeutic use. Second, dietarily derived LCFAs, including ALA, have a negative association with COVID-19 severity, with serum concentrations correlating to the severity of the disease [22,30]. The finding that ALA can bind to CoV-2 S and RSV-F1 is consistent with previous findings, where other free fatty acids were demonstrated to bind viral surface proteins and inhibit virus–host interactions [28,30]. Additionally, previous work has shown the potential of ALA binding to the CoV-2 major protease (MPro), compromising its activity [72]. Importantly, these fatty acids are collectively well tolerated and have a robust safety profile [73,74,75], and are amenable to developing broad antivirals. Our results showed significant synergy against CoV-2 with 200 μM acetate, which is >5-fold lower than plasma concentrations of acetate and 100 μM ALA levels, i.e., 10-fold lower than that commonly used in dietary supplements, suggesting that the concentrations producing synergy should be well tolerated. Interestingly, this phenotype was also seen in RSV-infected A549 cells treated with acetate and ALA, indicating that this approach may have broad-spectrum potential against respiratory viruses.

Another significant finding is our ability to incorporate ALA into the backbone of a liposomal nanoformulation, demonstrating that ALA triggers the breakdown of NPs upon contact with CoV-2. Using calcein as a surrogate for acetate in the formulation of ALA–liposomes, the results show a release of the payload upon viral interaction in vitro. For biodistribution studies, DIR was incorporated into the ALA–liposome-encapsulating acetate for tracking the liposome in in vivo studies. The results demonstrated the release of acetate payload in the lungs of mice inoculated with UV-inactivated virus, which continued for at least 8 h in the lung tissues (constituting the primarily affected organ). Thus, formulation of a novel nanosystem, i.e., the ALA liposome-encapsulating acetate which displayed an enhanced half-life, as well as retention in the lungs for an extended period of time, is critical to the success of this broad antiviral. However, the results of this part of the study in particular are limited due to the virus being administered being UV inactivated. Investigating how biodistribution could be impacted using replication-competent SARS-CoV-2 or other respiratory viruses of concern is warranted for future study. Also, the mechanism of virus induced rapid disintegration of NPs remains unclear. 

**Figure 7:**
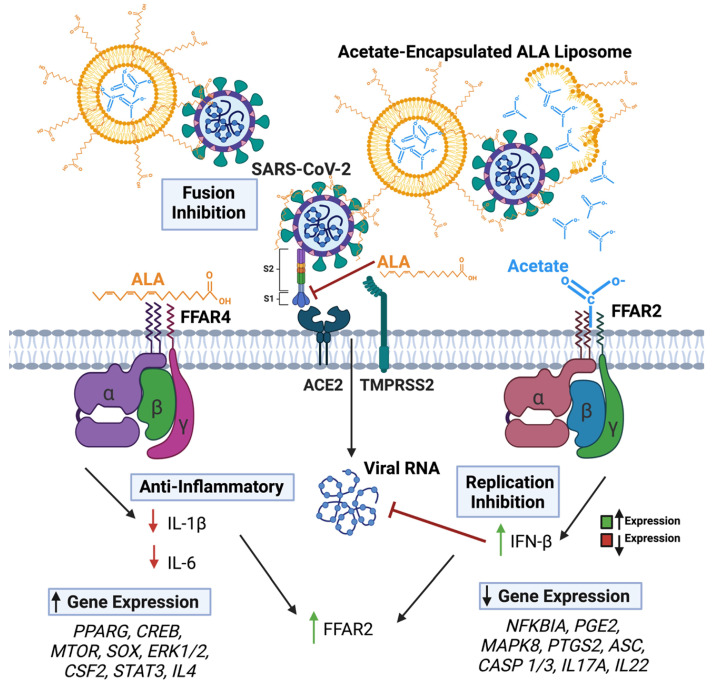
Graphical representation of SARS-CoV-2 and lipo + AL impacting FFAR activation and viral infection. Arrows in text boxes predicted up (left) and down (right) regulation of gene expression as predicted by IPA analyses, respectively due to FFAR4 (left) and FFAR2 (right) signaling.

It is also noteworthy that the superior effectiveness of the ALA–acetate liposomal NPs in vivo was confirmed in mice infected with MA10 in as far that the treatment significantly maintained body weight, showed a significant reduction in plaque counts from lung lysate, decreased lung histopathology, increased IFN-β expression, and decreased IL-1β and IL-6 expression. Our results showed a >3-log reduction in viral titers, which is comparable to other specific antivirals currently used in standard of care [76,77]. The mechanism behind the synergy between ALA and acetate is currently under further investigation and may involve crosstalk at the level of signaling between FFAR2 and FFAR4, which is schematically shown in Figure 7. First, the higher potency of therapeutic efficacy is due to the combination of inhibitory mechanisms involving viral fusion and entry into the cells and the inhibition of viral replication. Although the antiviral efficacy of ALA and acetate has been shown for only CoV2 and RSV in this report, it is expected that the effects can be extended to the influenza virus. For the latter, it has been reported that acetate can inhibit viral replication [12]. The second level of synergy appears to be in the levels of antiviral and anti-inflammatory mechanisms mediated by FFAR2 and FFAR4, respectively. To this end, an IPA analysis showed that the signaling through these receptors can lead to the expression of genes that may reciprocally regulate each other; for example, IL-4 produced by ALA–FFAR4 signaling, may regulate IFNs produced by FFAR2 signaling (Supplementary Figure S5). Thirdly, there is a possibility that FFAR2 and FFAR4 can interact to form a heteromer, which has been demonstrated by other studies to enhance cytosolic calcium signaling and β-arrestin-2 recruitment [78]. It is to be noted that these mechanisms are not mutually exclusive. Further investigation on the relationship of FFAR2 and FFAR4 is warranted and may yield a greater understanding of how these two receptors may impact one another, as well as their combined phenotypic alterations to epithelial and immune cells during infection or other diseased states.

While this work has demonstrated the synergy between acetate and ALA and the nanoformulations thereof in inhibiting CoV-2 and RSV infections at the cellular level, this needs to be extended to in vivo studies and other viruses to define its scope. Additionally, further mechanistic study needs to be undertaken regarding the FFAR2 and FFAR4 regulation and proposed crosstalk induced by treatment with acetate-ALA liposomes. Future work will focus on the downstream signaling mechanisms induced by FFAR2 and FFAR4 and defining the potential crosstalk relationship. Interestingly, other FFARs have previously been demonstrated to have this proposed relationship with one another [78]. Similarly, the scope of the ALA–liposome NPs may be reconfigured to add additional inhibitory small molecules and RNA-based drugs, such as the siRNAs or the micro-RNAs, for inclusion in the future formulation of NPs and showing potential broad applications for other respiratory viruses of interest. In addition, further mechanistic studies, time-course experiments, and therapeutic studies involving short-, medium-, and long-chain fatty acids are warranted, particularly those involving FFAR interrelation with one another [18].

In conclusion, our results demonstrate that acetate and ALA synergistically reduce respiratory viral infections caused by CoV-2 or RSV. Also, our results show that an ALA liposomal nanoformulation carrying acetate as an antiviral payload can release its payload upon interacting with CoV-2 and reduce Cov-2 infection and lung pathology. Furthermore, the results of studies on this mechanism underlying synergy appear to involve crosstalk between their receptors, i.e., FFAR2 and FFAR4, affecting each other’s expression and signaling functions.

## Data Availability

All other data generated or analyzed during this study are included in this published article (and its supplemental information).

## Supplementary Materials

The following supporting information can be downloaded at: https://www.mdpi.com/article/10.3390/v15071429/s1, Supplementary Table S1. List and sequences of qPCR Primers; Supplementary Figure S1: Linolenic acid binds to the receptor-binding domain of the SARS-CoV-2 spike protein, and RSV-F1; Supplementary Figure S2: Particle characterization of linolenic acid liposomes encapsulating acetate. Supplementary Figure S3: Cell viability assay of 3T3 (ATCC) and A549 (ATCC) Cells treated with ALA-acetate liposomes over a range of concentrations for 48 h; Supplementary Figure S4: FFAR2 Florescent-Immunohistochemistry Images; Supplementary Figure S5: Qiagen Ingenuity Pathway Analysis (IPA).
