# Supplementary material for: Acetate-encapsulated Linolenic Acid Liposomes Reduce SARS-CoV-2 and RSV Infection
Source: Viruses. 2023 Jun 24;15(7):1429. doi: 10.3390/v15071429 (PMC10385125; doi:10.3390/v15071429)
Supplement: Supplementary file 1 [file viruses-15-01429-s001.zip › viruses-2394493-supplementary.pdf]

Supplemental Data File:

Table of qPCR primers listed below. mm=mus musculus, hu = human.)

Supplementary Table S1. List and sequences of qPCR Primers:

Gene	Forward	Reverse
hu β-Actin	CACCATTGGCAATGAGCGGTTC	AGGTCTTTGCGGATGTCCACGT
mm β-Actin	CATTGCTGACAGGATGCAGAAGG	TGCTGGAAGGTGGACAGTGAGG
CoV2-N	CACATTGGCACCCGCAATC	GAGGAACGAGAAGAGGCTTG
mm FFAR2	CCACTGTATGGAGTGATCGCTG	GGGTGAAGTTCTCGTAGCAGGT
mm FFAR4	GTGACTTTGAACTTCCTGGTGCC	CAGAGTATGCCAAGCTCAGCGT
hu GAPDH	GTCTCCTCTGACTTCAACAGCG	ACCACCCTGTTGCTGTAGCCAA
mm GAPDH	CATCACTGCCACCCAGAAGACTG	ATGCCAGTGAGCTTCCCGTTCAG
hu IFN- β	CTTGGATTCCTACAAAGAAGCAGC	TCCTCCTTCTGGAACTGCTGCA
mm IFN- β	GCCTTTGCCATCCAAGAGATGC	ACACTGTCTGCTGGTGGAGTTC
hu IL-1β	CCACAGACCTTCCAGGAGAATG	GTGCAGTTCAGTGATCGTACAGG
mm IL-1β	TGGACCTTCCAGGATGAGGACA	GTTCATCTCGGAGCCTGTAGTG
hu IL-6	AGACAGCCACTCACCTCTTCAG	TTCTGCCAGTGCCTCTTTGCTG
mm IL-6	TACCACTTCACAAGTCGGAGGC	CTGCAAGTGCATCATCGTTGTTC

Linolenic acid binds to the receptor-binding domain of the SARS-CoV-2 spike protein, and RSV-F.1

Methods:

AutoDock Vina is an open-source program for performing molecular docking. The selection of the PDB:6M17for SARS CoV-2 was determined through accurate modeling of the ACE/RBD interaction of CoV-2 interaction facilitating viral entry. Receptors were prepared and configured using coordinate AutoGrid system implementing the Autodock 4.2 force field system. Likewise, the ligands were prepared following the same parameters configured to dock within the grid coordinates. Both ligand and receptor output files are PDBQT to execute Autodock Vina docking protocol to obtain appropriate scoring functions. Docking output of AutoDock Vina were imported to Pymol (Schrodinger) for evaluation and analysis of meaningful binding affinity. PDB:SVSB for NL63-CoV-S, and PDB: 4MMS RSF-F1 were used.

To examine binding of linolenic acid and SARS-CoV-2 virus, the TEM grids were prepared with 50uL of 400mg/mL ALA liposomes and were incubated with 50uL of 4x10^{8} pfu CoV-2 at room temperature for 30 minutes and then 50uL was applied to Lacey Carbon Supported Copper Grids size 300 mesh, size 100 nm (Fisher). Grids were washed and dried overnight and them imaged using a JEOL 1400 Transmission Electron Microscope. Liposomes alone and CoV-2 alone were also shown.

Results:

Docking studies also that both ALA and linoleic acid interact with CoV-2 S at the Arg 403, Gly 404, Asp 405, Glu 406 residues; albeit ALA binds more strongly to Arg 403 resulting in a higher ligand affinity score suggesting that ALA can act as a potent fusion inhibitor. The TEM analyses showed binding of the Linolenic acid liposomes to the SARS-CoV-2 virus.

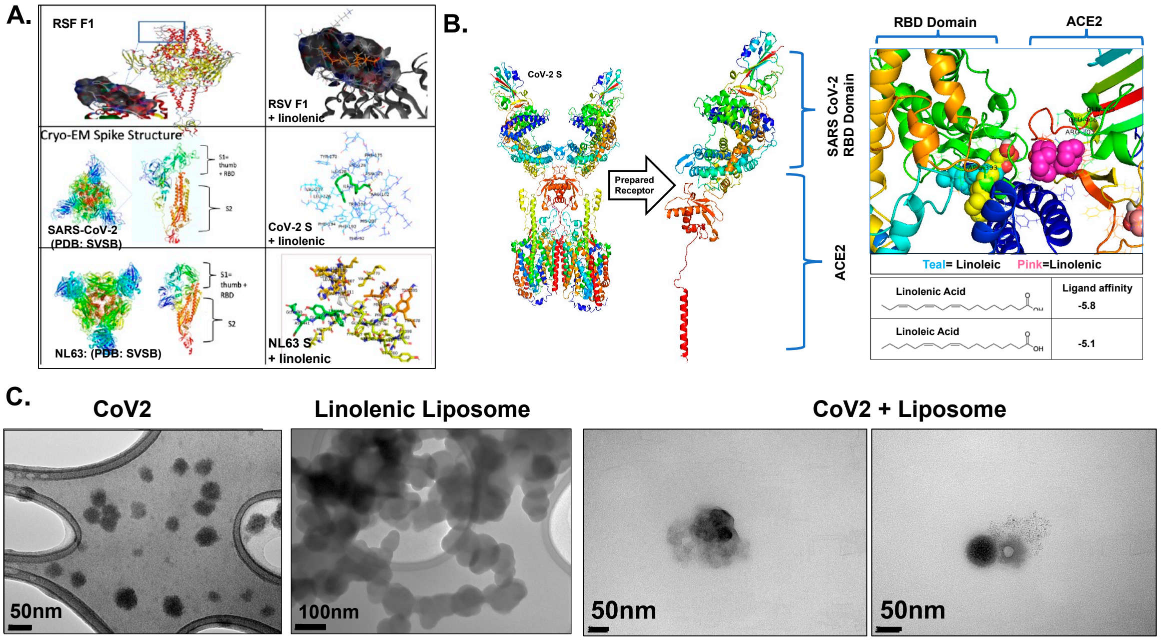

Supplementary Figure S1: Linolenic acid binds to the receptor-binding domain of the SARS-CoV-2 spike protein, and RSV-F1. A. A. Cryo-EM structure of the Fusion / Spike protein of SARS-CoV-2 and NL63-CoV and RSV F1 bound to linolenic acid B. SARS-CoV-2 S1 protein PDB: 6M17 (RBD: ACE2:B^{o}AT1) and AutoDock Vina analysis of linolenic and linoleic acid spatial position on the SARS-CoV-2 Spike – ACE2 interface with ligand affinity score. C.

Representative images of Transmission Electron Microscopy (TEM) of SARS-CoV-2, linolenic acid liposomes, and CoV2 – Liposome mixture

Supp Fig S2.

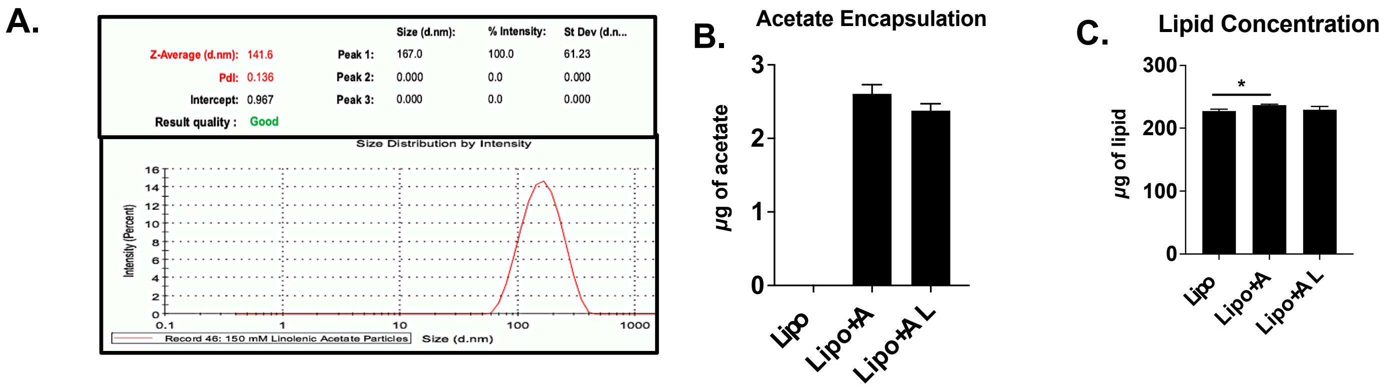

*Supplementary Figure S2: Particle characterization of linolenic acid liposomes encapsulating acetate. A. Average size of Linolenic acid liposome encapsulating acetate. B, C. concentration of acetate and lipids in liposome from a 50μL Sample. acetate was assayed using the Acetate Colorimetric Assay Kit (Sigma-Aldrich), and lipids were assayed using the Stewart assay method. Significance was determined using a one-way ANOVA with multiple comparisons, n=3, ± SD, p= *0.05.*

Supp Fig S3.

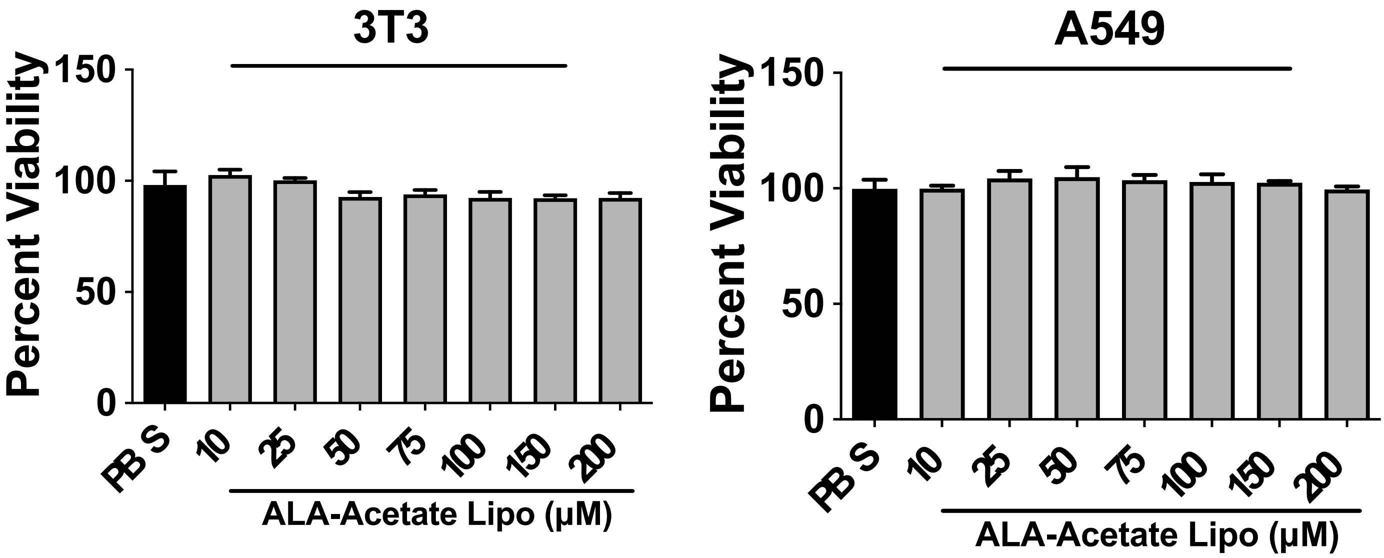

*Supplementary Figure S3: Cell viability assay of 3T3 (ATCC) and A549 (ATCC) Cells treated with ALA-acetate liposomes over a range of concentrations for 48 hours. Following the manufacturers protocol, cell titer glow 2.0 (Promega) was used to quantify cell viability. Significance was determined using a one-way ANOVA with multiple comparisons, n=4, ± SD, p= *0.05.*

Supp Fig S4.

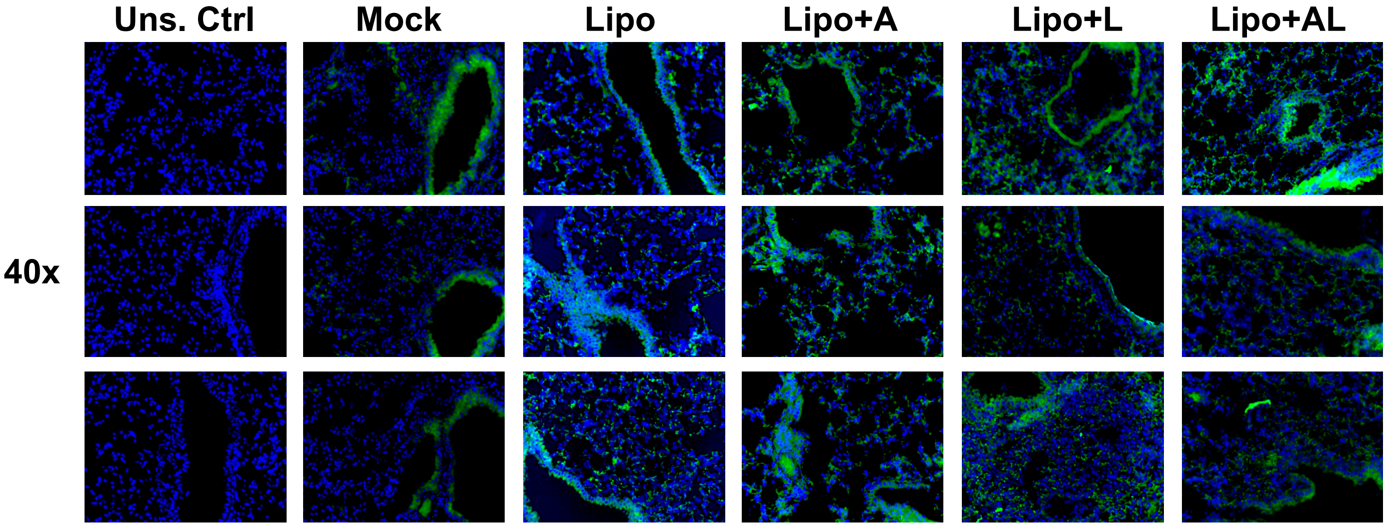

Supplementary Figure S4: FFAR2 Florescent-Immunohistochemistry Images. 10µm sections from paraffin embedded lungs processed for IHC and stained with anti-FFAR2 antibody conjugated to FITC, imaged at 40X.

Supp Fig S5.

Qiagen Ingenuity Pathway Analysis (IPA):

Ingenuity Pathway Analysis (IPA) (Qiagen) is a molecular pathway analysis application used for the analysis, integration and interpretation of data derived from curated 'omics experiments. IPA allowed for the prediction downstream effects and identify of candidate biomarkers. IPA was used to plot known connections between the genes creating a molecular network, with predicted up and downregulation. RNA-seq profiling of tissue samples expressing Free Fatty Acid Receptors 2 and 4 (FFAR 2/4) was used to formulate this network analysis.

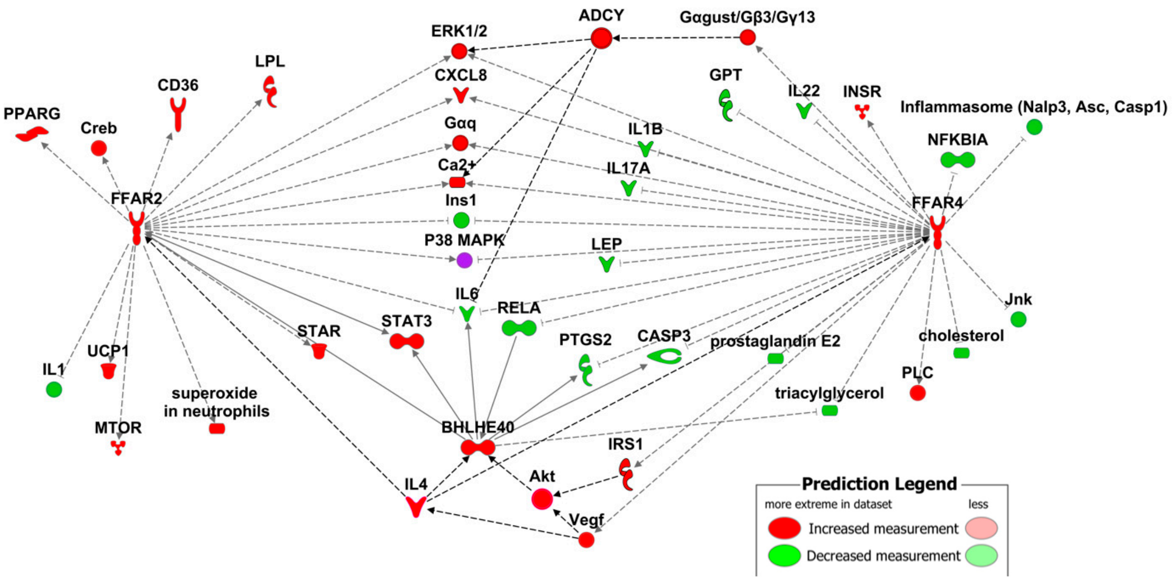

CrossTalk between FFAR2 and FFAR4:

FFAR2 and FFAR3 are expressed by alveolar macrophages, neutrophils, and AT2 cells. FFAR4 is also expressed by alveolar macrophages and to a lesser extent, club cells. We have observed an increase in FFAR2 and FFAR4 expression in the lungs of mice treated with acetate and linolenic acid (ligands of FFAR2 and FFAR4) but the mechanisms regulating their expression as a consequence of activation are poorly understood. Both FFARs are GPCRs with FFAR2 signaling through G1 and Gq to activate ERK, decrease cAMP, and increase intracellular calcium via phospholipase C. FFAR4 signaling also leads to ERK activation and calcium increase but it is also known to inhibit pro-inflammatory signaling through the TAB1/TAK1 pathway and activate AKT via PI3K.

For example. the cytokine interleukin 4 may play a central role in the crosstalk between FFAR pathways. IL-4 treatment is known to increase upregulation of FFAR2 in neutrophils. FFAR2 stimulation can upregulate IL-4 through MTOR and PPAR gamma. Mouse IL-4 has been shown to be necessary for FFAR4 expression in CD4+ TILs. Furthermore, FFAR2 stimulation can upregulate the expression of mitochondrial uncoupling protein UCP1 which regulates fatty acid metabolism and can thus lead to FFAR4 activation. In turn, FFAR4 upregulates Vegf and IL4 production stimulating FFAR2.
